# lncRNA MAGI2‐AS3 overexpression had antitumor effect on Hepatic cancer via miRNA‐23a‐3p/PTEN axis

**DOI:** 10.1002/fsn3.2199

**Published:** 2021-03-23

**Authors:** Fei Liu, Wenwen Deng, Zhenda Wan, Dajin Xu, Jun Chen, Xin Yang, Jianhua Xu

**Affiliations:** ^1^ Jiangxi Province Hospital of Integrated Chinese and Western Medicine Nanchang China

**Keywords:** Bel‐7402, Huh‐7, MAGI2‐AS3, miRNA‐23a‐3p, PTEN

## Abstract

The present study aimed to evaluate the antitumor effects of MAGI2‐AS3 and its mechanism in liver cancer. Cancer tissues and adjacent nontumor tissues were collected, and lncRNAs were analyzed *via* chip assay. The correlation between MAGEI2‐AS3 and patient pathology and prognosis was then analyzed. Bel‐7402 and Huh‐7 cell lines were also used in our study. For the in vitro study, MTT assay, flow cytometry, transwell assay, and wound healing assay were conducted to evaluate hepatic cancer cell (Bel‐7402 and Huh‐7) proliferation, apoptosis, invasion, and migration. The relative mechanisms were evaluated by Western blot (WB) and cellular immunofluorescence. The correlation among MAGI2‐AS3, miRNA‐23a‐3p, and PTEN was determined by a dual‐luciferase reporter assay. The expression of lncRNA MAGI2‐AS3 was significantly downregulated in tumor tissues. MAGI2‐AS3 expression was closely correlation with HCC patient's clinicopathology and prognosis and prognosis. In the cell experiment, compared with the negative control (NC) group, MAGI2‐AS3 overexpression reduced cell proliferation, invasion, and migration and increased cell apoptosis in Bel‐7402 and Huh‐7 cell lines. However, when Bel‐7402 and Huh‐7 cells were transfected with miRNA‐23a‐3p, their biological activities (proliferation, invasion, and migration) were significantly increased. Through WB assay, MAGI2‐AS3 could increase PTEN and depress p‐AKT and MMP‐9 protein expressions *via* miRNA‐23a‐3p suppression. The dual‐luciferase reporter assay revealed that MAGI2‐AS3 directly targeted miRNA‐23a‐3p and that miRNA‐23a‐3p could target PTEN. MAGI2‐AS3 might be a potential therapeutic target for liver cancer owing to its regulation by the miRNA‐23a‐3p/PTEN axis.

## Graphical abstract

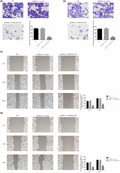

## 1 INTRODUCTION

More than 90% of the human genome is the transcriptome (Carninci et al., 2005; Kapranov et al., 2007; Willingham & Gingeras, 2006), which contains approximately 20,000 protein‐coding genes accounting for fewer than 2% of the entire genome (Wilusz et al., 2009). Due to the substantial progress in next‐generation sequencing techniques, thousands of lncRNAs can be quickly identified in vertebrates and invertebrates. The vast majority of lncRNAs are located in the nucleus, and increasing efforts have been made to explore their physiological and pathological functions. With a wide range of functions, lncRNAs are involved in various aspects of gene expression, translation and stabilization of proteins, as well as cell differentiation and organ formation. They are also involved in the pathological changes that are closely related to a variety of diseases, such as malignant tumors, cardiovascular diseases, and endocrine diseases. Although some lncRNAs are produced by RNA polymerase Ⅲ, most of them, such as coding genes, are transcribed by RNA polymerase Ⅱ (Dieci et al., 2007; Martone et al., 2003). Since RNA polymerase Ⅱ is located in the nucleus (Roeder & Rutter, 1970), lncRNAs are primarily produced in that location. However, unlike coding RNAs, lncRNAs do not contain an open reading frame and thus do not encode proteins. Depending on the relative position of lncRNAs and the protein‐coding genes on the chromosome, about half of the lncRNAs in the human genome are long intergenic noncoding RNAs (Hon et al., 2017). LncRNAs differ from mRNAs in terms of abundance, genome location, subcellular location, metabolic characteristics, epigenetic regulation, and tissue specificity (Ransohoff et al., 2018). LncRNAs are typically distributed in the nucleus, chromatin, or subnuclear region but are rarely found in the cytoplasm (Kretz et al., 2013). Compared with mRNAs, lncRNAs are associated with lower synthesis, higher degradation, and slower splicing (Ransohoff et al., 2018). LncRNAs are also more restricted to specific cell types than mRNAs. They have evolutionary conserved functions, secondary structures, and micro‐homologous regions, but there is a slight overall sequence similarity (Hezroni et al., 2015). Moreover, some studies found that lncRNA is correlated with the development of hepatocellular carcinoma (HCC) (Wang et al., 2017; Xiong et al., 2017).

As one of the most commonly diagnosed malignant tumors, liver cancer has high morbidity and mortality rates and thus seriously threatens human health. Moreover, it is associated with high medical costs and social burdens. Currently, approaches for the early diagnosis of liver cancer are quite limited and result in unfavorable treatment and prognosis. With the advances in next‐generation sequencing techniques, researchers have gradually found that noncoding RNAs in the human body play a significant role. LncRNAs have been confirmed to be closely related to the proliferation and invasiveness of liver cancer cells and to the prognosis of patients with liver cancer. They can function as oncogenes to induce liver cancer and to accelerate the growth and metastasis of liver cancer cells (Jiao et al., 2019; Zhang et al.,). In this study, a detection chip was used to evaluate lncRNA expression in liver cancer tissues depending on TCGA database, and patients who exhibited the largest difference were chosen as the research subjects. The role of lncRNAs in the biological activity of liver cancer cells and their underlying mechanisms were investigated *via* cell experiments.

## 2 MATERIALS AND METHODS

### 2.1 Case data

A total of 40 HCC patients who underwent radical resections for liver cancer from March 2011 to March 2013 at Jiangxi Integrated Chinese and Western Medicine Hospital and who satisfied the inclusion criteria were randomly selected. The inclusion criteria were as follows: (a) no other anticancer treatment received before surgery; (b) availability of complete clinical data; and (c) lesion pathologically confirmed as primary liver cancer following surgery. During the surgery, the liver cancer tissues and adjacent liver tissues (distance from tumor boundary > 2 cm with pathologic confirmation of no tumor cells) were collected and stored at −80°C for later use. All patients provided informed consent, and approval from the hospital's ethics committee was obtained.

Adjacent nontumor tissues from six cases and liver cancer tissues from five cases were randomly selected for the chip assay. Chip test and lncRNA differential analysis were conducted by Shanghai GeneChem Co., Ltd.

### 2.2 Follow‐up

Patients were followed up regularly to determine their prognosis. Short‐term follow‐up (within 1 year) was mainly conducted through outpatient review, whereas long‐term follow‐up (beyond 1 year) was mainly conducted through telephone interview. The patients were followed up at an interval of 3 months for the first 2 years after surgery and at an interval of 6 months after 2 years. The follow‐up ended on March 2015, and the median follow‐up time was 45.3 (range, 1–60) months; no patients were lost to follow‐up. The follow‐up covered postoperative prognosis (progression, recurrence, and death), imaging changes, blood AFP levels, and liver function.

### 2.3 LncRNA microarray screening

Adjacent nontumor tissues (*n* = 6) and cancer tissues (*n* = 5) were subjected to microarray detection and analyzed by Shanghai GeneChem Co., Ltd.

### 2.4 RT‐qPCR assay

According to the manufacturer's instructions, total RNAs were routinely extracted using TRIzol reagent (Toyobo, Osaka, Japan). Reverse transcription reactions were performed according to the manufacturers’ protocol. The total reaction system was 20 μl, including 2 μg of sample RNA, 1 μl of primer mix (0.5 μl forward primer and 0.5μL reverse primer), 4 μl of 5 × buffer, 1 μl of enzyme, and 12 μl Diethyl pyrocarbonate (DEPC). The reaction was performed at 37°C for 15 min, followed by inactivation at 98°C for 5 min. Glyceraldehyde‐3‐phosphate dehydrogenase (GAPDH) served as the internal control for MAGI2‐AS3, whereas U6 served as the internal control for miRNA‐23a‐3p. MAGI2‐AS3, GAPDH, miRNA‐23a‐3p, and U6 were detected by relative fluorescence quantitative PCR, and the relative expression levels of the target genes were calculated for different samples. The primer sequences are presented in Table 1.

**TABLE 1:** The primer sequences

Gene name	Primer sequences
MAGI2‐AS3	F: 5′‐CACCTTGCTTGACACACTTGA−3′
	R:5′‐CATTACAGCTCGGCTACTGC−3′
miRNA−23a−3p	F: 5′‐GCGATCACATTGCCAGGG−3′
	R: 5′‐CAGTGCGTGTCGTGGAGT−3′
U6	F:5′‐CTCGCTTCGGCAGCACA−3′
	R:5′‐AACGCTTCACGAATTTGCGT−3′
GAPDH	F:5′‐AGGTCGGTGTGAACGGATTTG −3′
	R: 5′‐GGGGTCGTTGATGGCAACA−3′

### 2.5 Cell culture and transfection

L02 (normal hepatic cells) and the hepatocellular carcinoma cells Bel‐7402, Huh‐7, HepG2, and SMMC‐7721 (ATCC, USA) were used. L02, Bel‐7402, Huh‐7, HepG2, and SMMC‐7721 cells were cultured in RPMI 1,640 medium supplemented with 10% fetal bovine serum (FBS) contained 100 μg/ml penicillin and 0.1 mg/ml streptomycin in a 5% CO_2_ incubator at 37°C. The culture medium was replaced with fresh medium every 2 days. When the cell adherent growth rate reached 80% or more, trypsin was used for digestion and passage. Bel‐7402 and Huh‐7 cells in the logarithmic growth phase were selected and inoculated into a 96‐well plate at 5 × 10^5^ cells/well. Then, the cells were incubated in a 37°C constant temperature incubator. Transfection was conducted when cells were 50% confluent. The transfection process was performed using Lipofectamine 2000 (Invitrogen, Carlsbad, CA, USA), according to the manufacturer's instructions. The pcDNA3.1‐vector, miRNA‐NC, MAGI2‐AS3, and miRNA‐23a‐3p were synthesized by NanJing KeyGen Biotech Co., Ltd. Subsequent experiments were conducted 48 hr after transfection.

### 2.6 MTT assay

Cells in all groups were inoculated into a 96‐well plate at a density of 4 × 10^3^ cells/well. At 72 hr after transfection, the cells from each group were removed from the 6 wells, and 20 µl of the MTT solution was added. Subsequently, the plates were incubated in an incubator at 37°C for 4 hr. After the cell culture plate was removed, 100 μl of dimethyl sulfoxide was added to the samples, which were then oscillated for 10 min. The cell absorbance value (A value) at 450 nm was detected using a microplate reader.

### 2.7 Apoptosis experiment

Cells of all groups were treated for 48 hr and then digested and collected to generate a single‐cell suspension. The cells were washed twice with cold PBS solution. Binding buffer was then diluted with deionised water at a ratio of 1∶4 (20 ml binding buffer + 60 ml deionized water). The cells were resuspended in 250 μl of diluted binding buffer, and the cell density was adjusted to 1 × 10^6^ cells/ml. Cell suspension (100 μl) was placed in a 5‐ml flow tube, at which point 5 μl of Annexin V and 10 μl of PI dye were added, followed by staining at room temperature in the dark for 15 min. No Annexin V was added to the control tube. Next, 400 μl of binding buffer was immediately added to the flow tube for the flow cytometry. The light source was a 488‐nm argon ion laser; Annexin V was excited to emit green fluorescence, whereas PI emitted red fluorescence. The results were analyzed using random software.

### 2.8 Transwell invasion assay

The bottom of the Transwell chamber (with a well size of 8 μm) was coated with a 1∶8 dilution of 50 mg/L Matrigel and was air‐dried at 4°C. Then, 50 μl of serum‐containing RPMI 1640 medium was added (37°C, 30 min). After cells from all the groups were treated for 48 hr, the serum was removed, and the cells were serum‐starved for 12 hr. The cells were then digested and collected. After they were resuspended, the cells were counted and the cell suspension was adjusted to 1 × 10^6^ cells/ml. The Transwell chamber was placed in a 24‐well cell culture plate, and 200 μl of the cell suspension was added to the Transwell chamber. The number of cells was 2 × 10^5^. Then, 500 μl of the complete medium containing 100 ml/L FBS was added to the lower chamber of a 24‐well plate. The experiment was repeated using three samples in each group. After incubation in a 50 ml/L CO_2_ incubator at a constant temperature of 37°C for 48 hr, the cells were removed, fixed in 950 ml/L ethanol, and stained with 1 g/L crystal violet. The cells were observed under an inverted microscope and imaged, and the number of cells that had migrated to the lower layer of the microporous membrane was counted. Five fields of view were randomly selected for each sample, and the average value was calculated.

### 2.9 Wound healing assay

Cells in the logarithmic growth phase were obtained from each group, enzyme‐digested and centrifuged and then resuspended as a single‐cell suspension, which was evenly inoculated into a 24‐well cell culture plate. After the cells were grown as a single layer, the culture solution was discarded. A 200‐μl pipette tip was used to draw a mark at the center of each well of the 24‐well plate. After the dead cells were washed away, the samples were imaged under a microscope and recorded at time point 0 hr. After drug treatment for 24 and 48 hr, the healing of the cell scratches at these 2 time points was recorded and imaged at the same observation points. This experiment was repeated three times. Image‐Pro Plus software was used to measure the multi‐scratch distance of each well, and the average value was calculated. Then, the distance after the treatment was subtracted from the distance before the treatment, the value of which was the cell migration distance for 24 subjects over 48 hr. The data were analyzed using statistical software.

### 2.10 Western blot assay for relative protein expression

The cells of all groups were collected 48 hr after transfection, washed twice with precooled PBS, and placed on ice for the extraction of total cell proteins. The protein concentration was determined *via* the BCA method, and 40 mg of protein was added to the sample wells with sodium lauryl sulfate. Polyacrylamide gel was used for electrophoresis, after which the cells were transferred to the membrane and blocked for 1 hr at room temperature in 5% skim milk powder. Primary antibodies against PTEN (50 kDa), phosphorylated AKT(68 kDa), MMP‐9 (94 kDa), and GAPDH(37 kDa)(Abcam, Cambridge, UK) were added. The dilution ratio was 1:1,000, and incubation was performed at 4°C overnight. Then, the membrane was washed with TBST for 3 times (10 min each time) and incubated with the appropriate secondary antibody (diluted 1:5,000) at room temperature for 2 hr. The membrane was washed with TBST for three times (10 min each time), incubated with ECL illuminant, and developed and imaged with a gel imaging system. The ImageJ analysis software was used to analyze the gray value of each band, which was calibrated with GAPDH, and the relative expression levels of the target proteins in the cells of all groups were calculated.

### 2.11 Cell immunofluorescence assay

Bel‐7402 and Huh‐7 cells were inoculated into a 24‐well plate containing glass coverslips. pcDNA3.1, miRNA‐NC, MAGI2‐AS3, and miRNA‐23a‐3p were transfected into the cells, which were observed after 48 hr. Cell immunofluorescence staining was employed to detect the localization of p‐AKT protein in the nucleus. Briefly, the steps were as follows: after cell growth, the samples were fixed in 40 g/L of paraformaldehyde solution and immediately treated with 2.5 ml/L of Triton X‐100 at room temperature for 10 min. After washing with PBS for 5 × 3 min, the cells were blocked in normal goat serum at room temperature for 30 min and incubated with a rabbit antihuman primary antibody against p‐AKT (1:100, Abcam, Cambridge, UK) at 4°C overnight. After washing with PBS for 5 × 3 min, the cells were incubated with an FITC‐labeled goat anti‐rabbit secondary antibody at room temperature in the dark for 2 hr. This experiment was repeated three times, and three duplicate wells were included for each group. The coverslips were sealed onto slides with glycerine and then observed and imaged using a laser confocal microscope (Nikon, Japan). Quantitative analysis was conducted on the results of the fluorescent staining.

### 2.12 Dual‐luciferase reporter assay

The 3'UTR fragment of MAGI2‐AS3 was cloned into a plasmid to construct a wild‐type MAGI2‐AS3‐WT reporter gene plasmid. The mutant MAGI2‐AS3‐Mut plasmid was constructed by mutating the miRNA‐23a‐3p binding site on the MAGI2‐AS3‐3'UTR using a Takara point mutation kit. The plasmid and miRNAs were transfected into the cells using Lipofectamine 2000, according to the manufacturer's protocols. Specifically, the cells transfected with the MAGI2‐AS3‐WT plasmid and miRNA‐23a‐3p mimics were classified as the MAGI2‐AS3‐WT group, whereas the cells co‐transfected with miRNA‐NC served as the control group. The cells transfected with the MAGI2‐AS3‐Mut plasmid and miRNA‐23a‐3p were classified as the MAGI2‐AS3‐Mut group, whereas the cells co‐transfected with miRNA‐NC served as the control group. After 48 hr, the cells were collected, and the luciferase activities of the cells in all groups were determined using a dual‐luciferase assay kit.

### 2.13 Statistical analysis

The experimental results were analyzed using the SPSS 22.0 software. The count data were analyzed by x^2^, and the measurement data were expressed as means ± *SD*. The Wilcoxon signed‐rank sum test was employed for the paired groups. Kaplan–Meier and log‐rank tests were employed for the survival analysis. A multivariate analysis was conducted using the Cox regression model. *p* <.05 was considered statistically significant.

## 3 RESULTS

### 3.1 Clinical data and analysis

**FIGURE 1:**
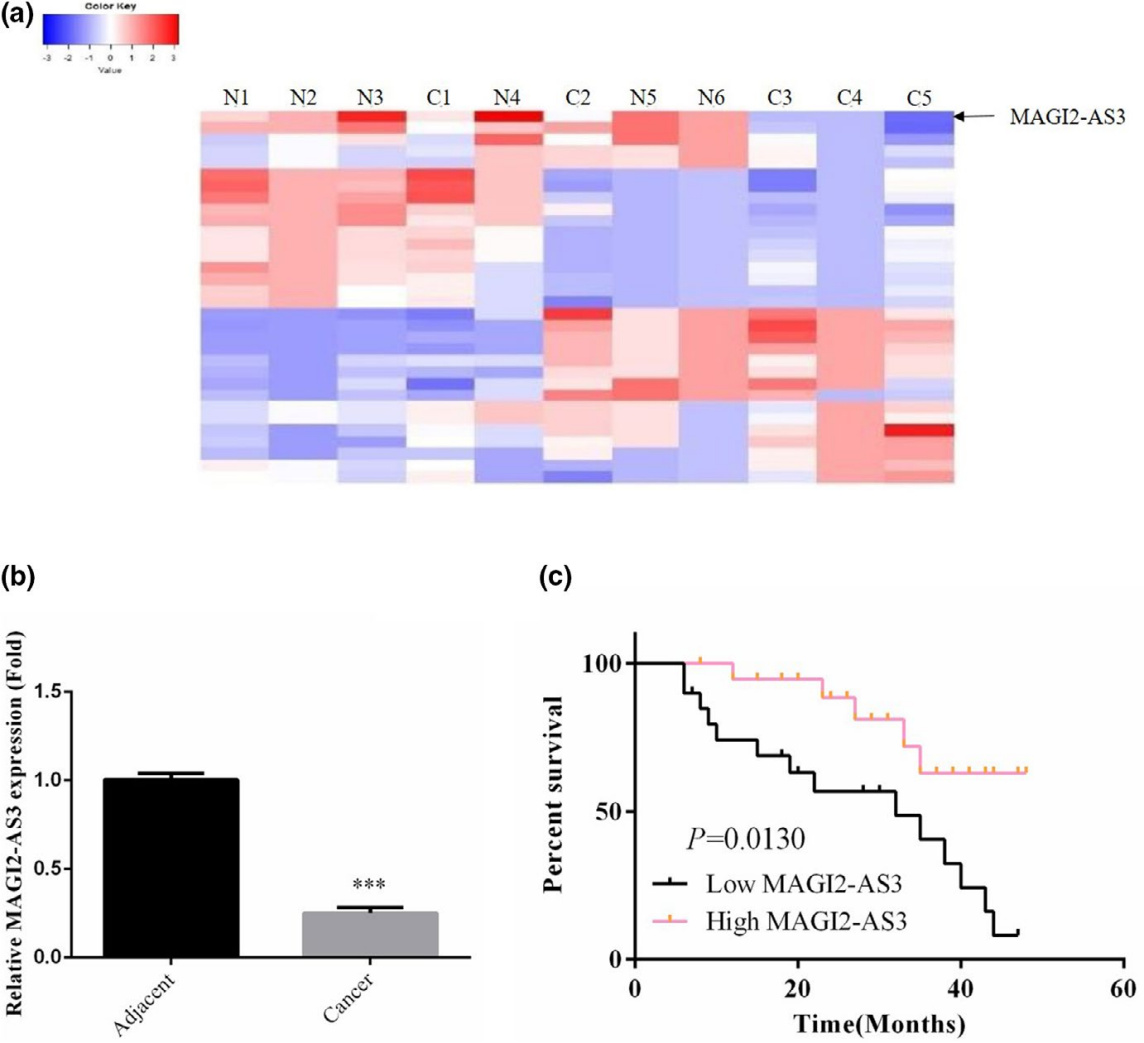
Clinical data and analysis. (a) Hot map. N: Adjacent nontumor tissues; C: liver cancer tissues. (b) MAGI2‐AS3 mRNA expression in difference tissues by RT‐qPCR assay. Adjacent: Adjacent nontumor tissues; Cancer: liver cancer tissues. ***: *p* <.001, compared with adjacent nontumor tissues. (c) Percent survival

A fold change ≥3.0 was considered to be the screening criterion for differentially expressed lncRNA genes. A heat map was used to show the expression levels of these genes in liver cancer tissues and adjacent nontumor tissues, as presented in Figure 1a. Among them, MAGI2‐AS3 exhibited the greatest difference, and thus, it was selected as the subject for this study. RT‐qPCR was used to determine the expression of MAGI2‐AS3 mRNA in adjacent nontumor tissues and in liver cancer tissues. The results revealed that the expression of MAGI2‐AS3 mRNA was significantly downregulated in liver cancer tissues (*p* <.001, Figure 1b). Based on the median of MAGI2‐AS3 mRNA expression in the tissue samples, the 40 patients were divided into the MAGI2‐AS3 low‐expression group and the MAGI2‐AS3 high‐expression group. A clinicopathologic analysis revealed that tumor size, TNM stage, and metastasis were significantly improved in the MAGI2‐AS3 high‐expression group compared with the MAGI2‐AS3 low‐expression group (*p* <.05, Table 2). The survival analysis revealed that the survival rate of patients in the MAGI2‐AS3 high‐expression group was significantly higher than that of patients in the MAGI2‐AS3 low‐expression group (*p* =.0130, Figure 1c).

**TABLE 2:** The correlation between MAGI2‐AS3 level and pathological characteristics of Hepatic cancer patients (*n* = 40)

Clinical pathologic features	Numbers	MAGI2‐AS3 expression		*p* value
		Low (*n* = 20)	High (*n* = 20)	
Age (year old)				
≤50	19	9	10	.613
>50	21	11	10	
Gender				
Male	22	12	10	.451
Female	18	8	10	
Tumor Size				
≤3 cm	16	4	12	.011
>3 cm	24	16	8	
TNM stage				
I‐II	15	4	11	.021
III‐IV	25	16	9	
Metastasis				
Yes	23	15	8	.033
No	17	5	12	

### 3.2 MAGI2‐AS3 gene expression and its effects on Bel‐7402 and Huh‐7 cell proliferation and apoptosis

**FIGURE 2:**
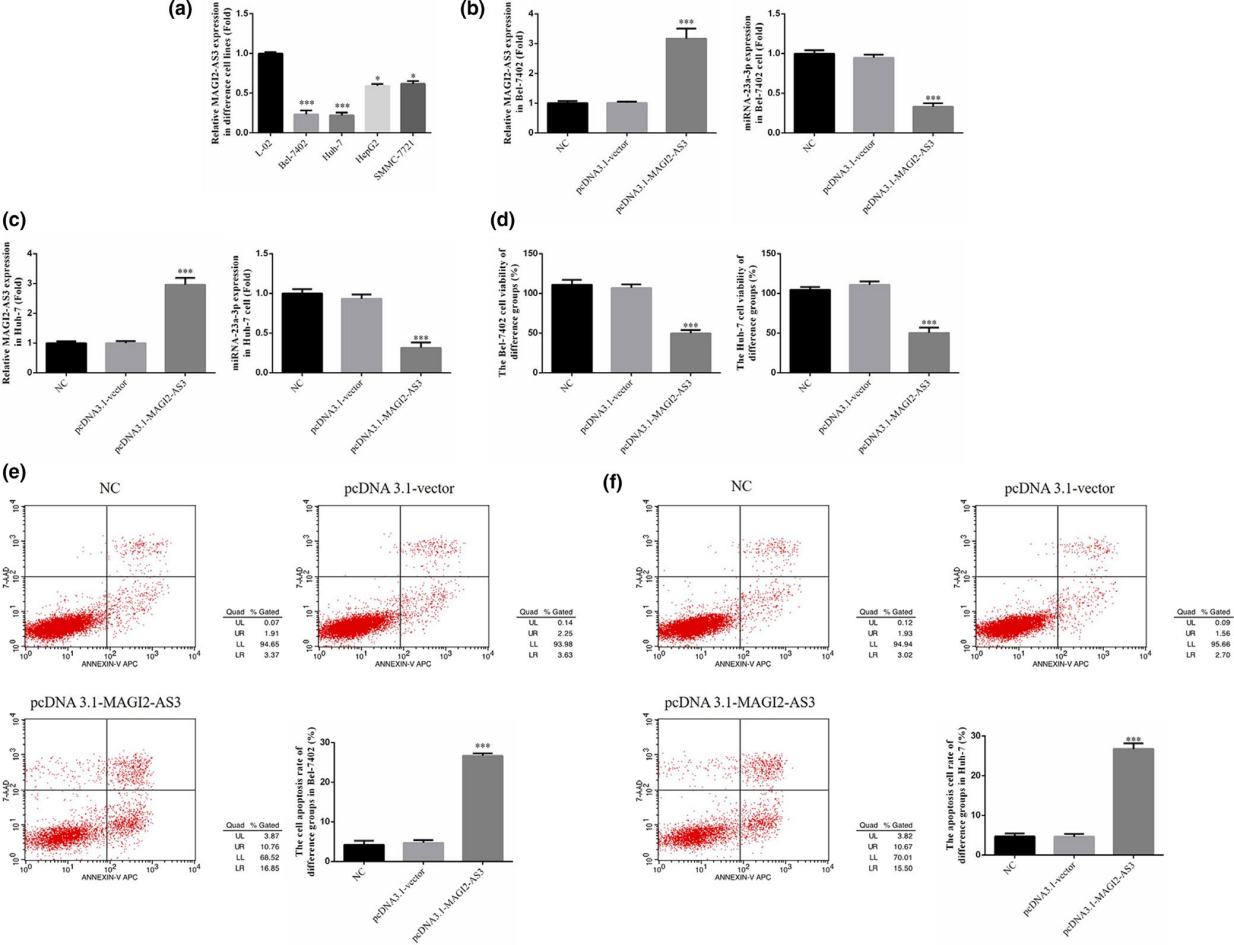
MAGI2‐AS3 Gene Expression and Its Effects on Bel‐7402 and Huh‐7 Cell biological activities. NC: Bel‐7402 cell were treated with normal; pcDNA3.1‐vector: Bel‐7402 cell were transfected with pcDNA3.1‐vector which were negative control; pcDNA3.1 MAGI2‐AS3: Bel‐7402 cell were transfected with pcDNA3.1 MAGI2‐AS3. (a) MAGI2‐AS3 gene expression in difference cell lines by RT‐qPCR assay. *: *p* <.05, ***: *p* <.001, compared with L‐02 cell line. (b) MAGI2‐AS3 and miRNA‐23a‐3p gene expression of difference groups in Bel‐7402 cell. ***: *p* <.001, compared with NC group. (c) MAGI2‐AS3 and miRNA‐23a‐3p gene expression of difference groups in Huh‐7 cell. ***: *p* <.001, compared with NC group. (d) The cell viability of difference groups in Bel‐7402 and Huh‐7. ***: *p* <.001, compared with NC group. (e). The cell apoptosis rate of difference groups in Bel‐7402 cell line by flow cytometry. ***: *p* <.001, compared with NC group. (f). The cell apoptosis rate of difference groups in Huh‐7 cell line by flow cytometry. ***: *p* <.001, compared with NC group

RT‐qPCR revealed that, compared with the normal liver cell line L‐02, the levels of MAGI2‐AS3 gene expression in Bel‐7402, Huh‐7, HepG2, and SMMC‐7721 liver cancer cells were significantly lower (*p* <.05, respectively). Among the liver cancer cell strains, Bel‐7402 and Huh‐7 cells exhibited the lowest level of MAGI2‐AS3 expression, as presented in Figure 2a. After the transfection of MAGI2‐AS3 into Bel‐7402 and Huh‐7 cells, RT‐qPCR revealed that the expression level of MAGI2‐AS3 m RNA was significantly increased, whereas that of miRNA‐23a‐3p mRNA was significantly decreased in the pcDNA3.1‐MAGI2‐AS3 group in these two cell types (*p* <.001, Figure 2b,c). The results of the MTT and flow cytometry assays revealed that the cell proliferation rate of Bel‐7402 and Huh‐7 cells in the pcDNA3.1‐MAGI2‐AS3 group was significantly decreased and that the apoptosis rate was significantly increased (*p* <.001, Figure 2d‐f).

### 3.3 Effects of MAGI2‐AS3 on Bel‐7402 and Huh‐7 cell invasiveness and migration

**FIGURE 3:**
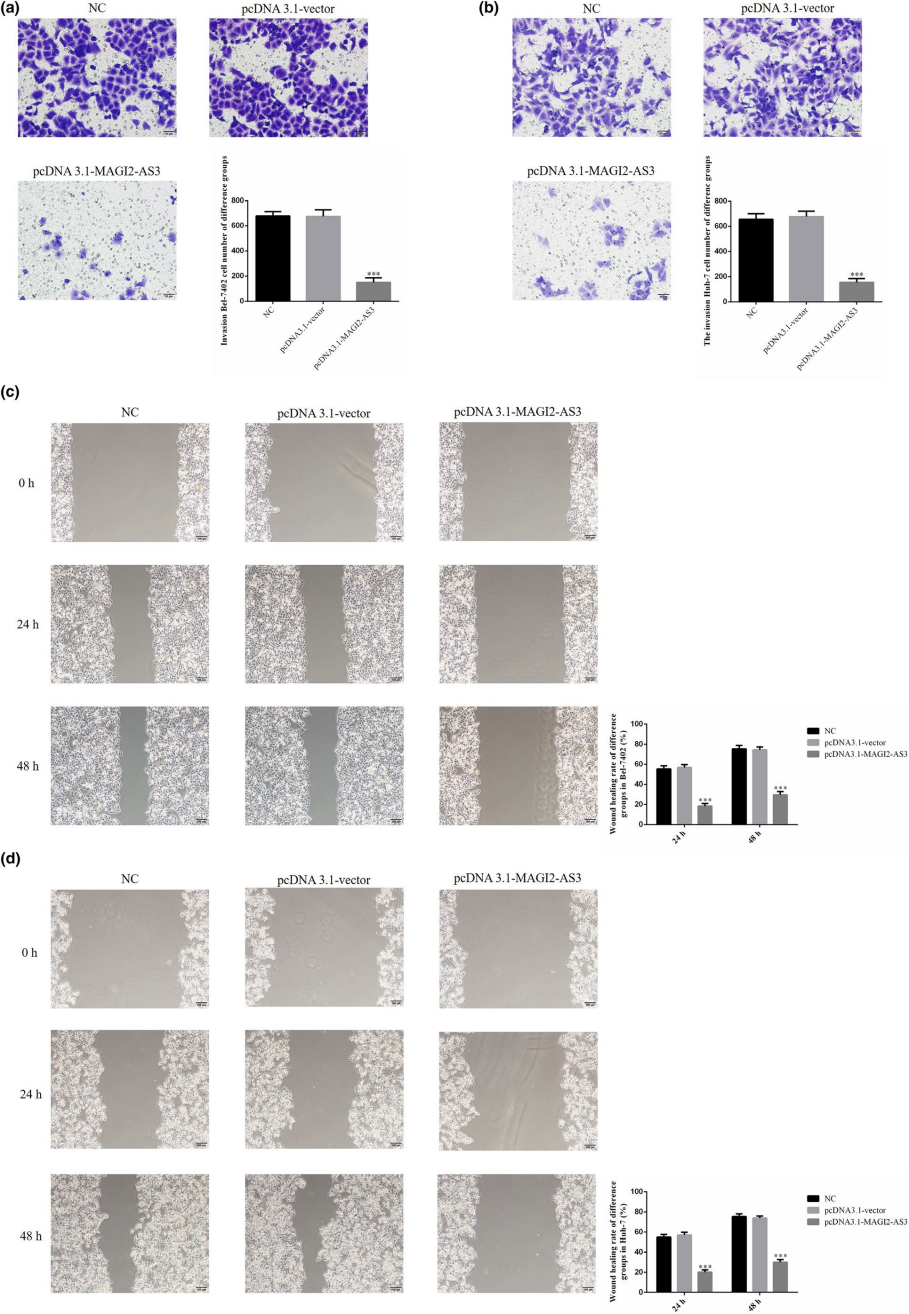
MAGI2‐AS3 had effects on cell invasion and migration. NC: Bel‐7402 cell were treated with normal; pcDNA3.1‐vector: Bel‐7402 cell were transfected with pcDNA3.1‐vector which were negative control; pcDNA3.1 MAGI2‐AS3: Bel‐7402 cell were transfected with pcDNA3.1 MAGI2‐AS3. (a) MAGI2‐AS3 affect invasion Bel‐7402 cell number of difference groups by transwell assay (200×). ***: *p* <.001, compared with NC group. (b) MAGI2‐AS3 affect invasion Huh‐7 cell number of difference groups by transwell assay (200×). ***: *p* <.001, compared with NC group. (c) MAGI2‐AS3 affect Bel‐7402 cell wound healing rate of difference groups (100×). ***: *p* <.001, compared with NC group. (d). MAGI2‐AS3 affect Huh‐7 cell wound healing rate of difference groups (100×). ***: *p* <.001, compared with NC group

To evaluate the effects of MAGI2‐AS3 on the invasiveness and migration of liver cancer cells, a Transwell assay was performed and revealed that in the pcDNA3.1‐MAGI2‐AS3 group of Bel‐7402 and Huh‐7 cells, the number of invading cells was significantly lower than that in the NC group (*p* <.001, respectively, Figure 3a,b). Moreover, a wound healing assay revealed that in the pcDNA3.1‐MAGI2‐AS3 group of Bel‐7402 and Huh‐7 cells, the wound healing rates at 24 and 48 hr were significantly lower than those in the NC group (*p* <.001, Figure 3c,d).

### 3.4 Effects of MAGI2‐AS3 on relative protein expression and p‐AKT nuclear localization

**FIGURE 4:**
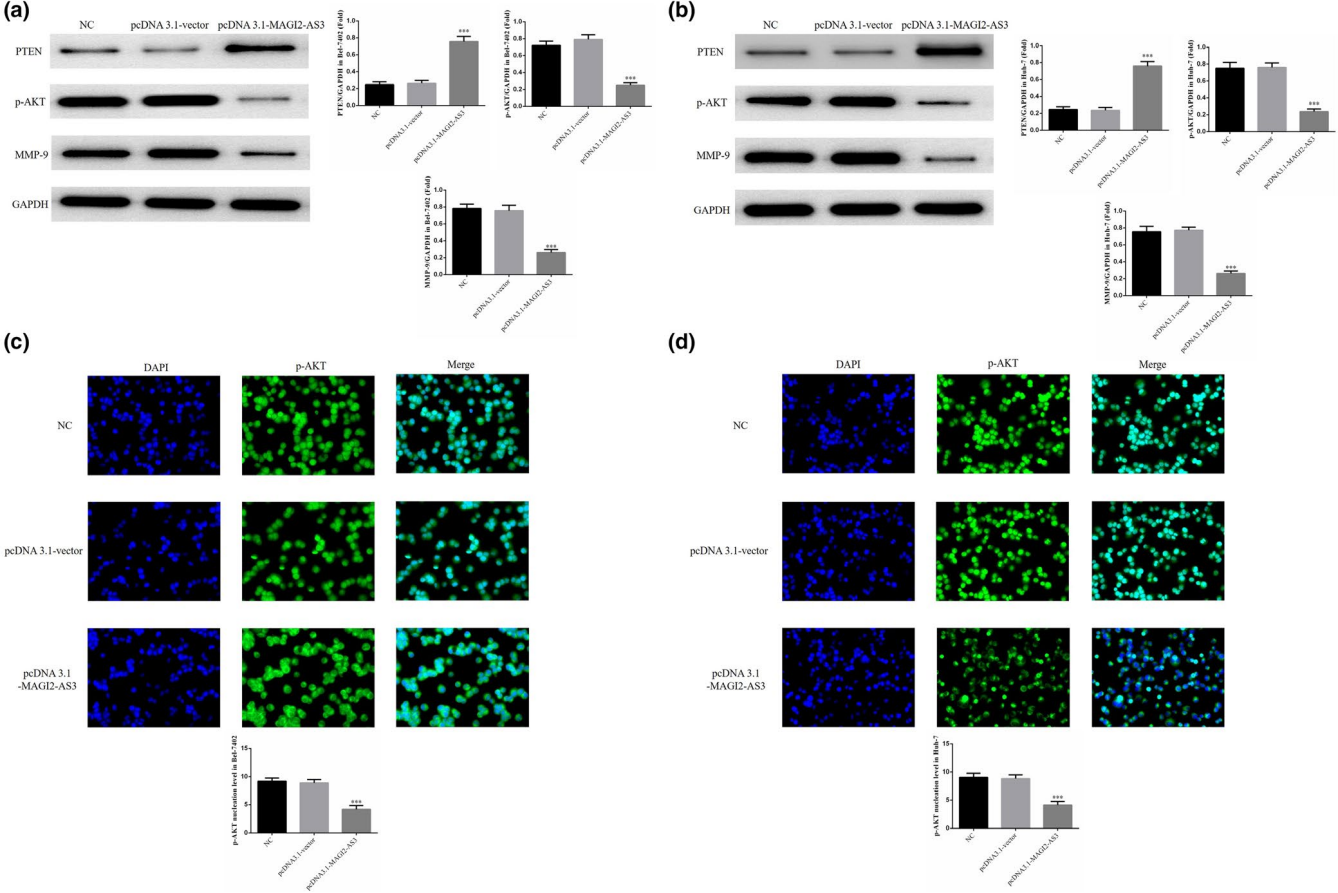
Effects of MAGI2‐AS3 on Related Proteins and p‐AKT Nucleation. NC: Bel‐7402 cell were treated with normal; pcDNA3.1‐vector: Bel‐7402 cell were transfected with pcDNA3.1‐vector which were negative control; pcDNA3.1 MAGI2‐AS3: Bel‐7402 cell were transfected with pcDNA3.1 MAGI2‐A (a) MAGI2‐AS3 affect relative proteins in Bel‐7402 by WB assay. ***: *p* <.001, compared with NC group. (b) MAGI2‐AS3 affect relative proteins in Huh‐7 by WB assay. ***: *p* <.001, compared with NC group. (c) MAGI2‐AS3 affect p‐AKT nuclear volume in Bel‐7402 cell. ***: *p* <.001, compared with NC group. (d) MAGI2‐AS3 affect p‐AKT nuclear volume in Huh‐7 cell. ***: *p* <.001, compared with NC group

WB was employed to determine the relative protein expression. In the Bel‐7402 and Huh‐7 cell lines, the expression of PTEN protein was significantly higher, whereas that of p‐AKT and MMP‐9 was significantly lower in the pcDNA3.1‐MAGI2‐AS3 group compared with the NC group (*p* <.001, Figure 4a,b). The cell immunofluorescence assay revealed that in Bel‐7402 and Huh‐7 cells, the nuclear localization of p‐AKT protein was significantly lower in the pcDNA3.1‐MAGI2‐AS3 group than in the NC group (*p* <.001, Figure 4c,d).

### 3.5 Expression levels of MAGI2‐AS3 and miRNA‐23a‐3p mRNA and effects of miRNA‐23a‐3p on Bel‐7402 and Huh‐7 cell proliferation and apoptosis

**FIGURE 5:**
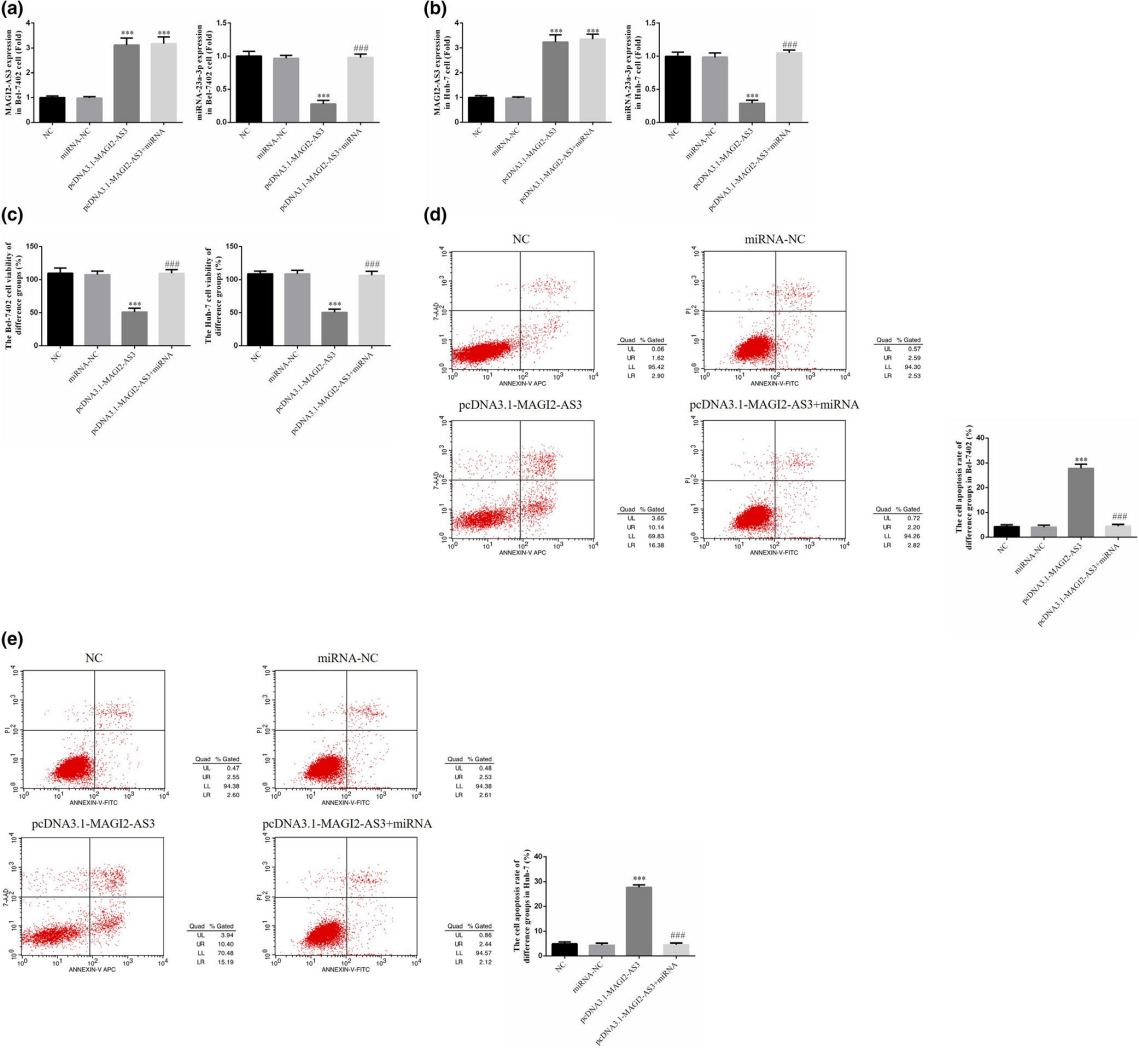
Expressions of MAGI2‐AS3 and miRNA‐23a‐3p mRNA and Effects of miRNA‐23a‐3p on Bel‐7402 and Huh‐7 Cell biological activities. NC: The cell were treated with normal; miRNA‐NC: The cell were transfected with miRNA‐negative control; pcDNA3.1‐MAGI2‐AS3: The cell were transfected with pcDNA3.1‐MAGI2‐AS3; pcDNA3.1‐MAGI2‐AS3 + miRNA: The cell were transfected with pcDNA3.1‐MAGI2‐AS3 and miRNA‐23a‐3p. (a) MAGI2‐AS3 and miRNA‐23a‐3p mRNA expression of difference groups in Bel‐7402 cell. ***: *p* <.001, compared with NC group; ###: *p* <.001, compared with pcDNA3.1‐MAGI2‐AS2 group. (b) MAGI2‐AS3 and miRNA‐23a‐3p mRNA expression of difference groups in Huh‐7 cell. ***: *p* <.001, compared with NC group; ###: *p* <.001, compared with pcDNA3.1‐MAGI2‐AS2 group. (c) miRNA‐23a‐3p affect cell viabilities by MTT assay in Bel‐7402 and Huh‐7 cell lines. ***: *p* <.001, compared with NC group; ###: *p* <.001, compared with pcDNA3.1‐MAGI2‐AS2 group. (d) miRNA‐23a‐3p affect Bel‐7402 cell apoptosis by flow cytometry. ***: *p* <.001, compared with NC group; ###: *p* <.001, compared with pcDNA3.1‐MAGI2‐AS2 group. (e) miRNA‐23a‐3p affect Huh‐7 cell apoptosis by flow cytometry. ***: *p* <.001, compared with NC group; ###: *p* <.001, compared with pcDNA3.1‐MAGI2‐AS2 group

In Bel‐7402 and Huh‐7 cells, the level of MAGI2‐AS3 m RNA expression was significantly increased in the pcDNA3.1‐MAGI2‐AS3 and pcDNA3.1‐MAGI2‐AS3 + miRNA groups compared with the NC group (*p* <.001, Figure 5a,b). The gene expression level of miRNA‐23a‐3p was significantly decreased in the pcDNA3.1‐MAGI2‐AS3 group (*p* <.001, Figure 5a,b). Compared with the pcDNA3.1‐MAGI2‐AS3 group, the expression level of miRNA‐23a‐3p mRNA was significantly increased in the pcDNA3.1‐MAGI2‐AS3 + miRNA group (*p* <.001, Figure 5a,b). MTT and flow cytometry assays revealed that in Bel‐7402 and Huh‐7 cells, the cell proliferation rate in the pcDNA3.1‐MAGI2‐AS3 group was significantly decreased, whereas the apoptosis rate was significantly increased compared with the NC group (*p* <.001, Figure 5c‐e). After the cells were co‐transfected with miRNA‐23a‐3p, the cell proliferation rate in the pcDNA3.1‐MAGI2‐AS3 + miRNA group was significantly increased, whereas the apoptosis rate was significantly decreased compared with the pcDNA3.1‐MAGI2‐AS3 group (*p* <.001, Figure 5c–e).

### 3.6 Effects of miRNA‐23a‐3p on Bel‐7402 and Huh‐7 cell invasion and migration

**FIGURE 6:**
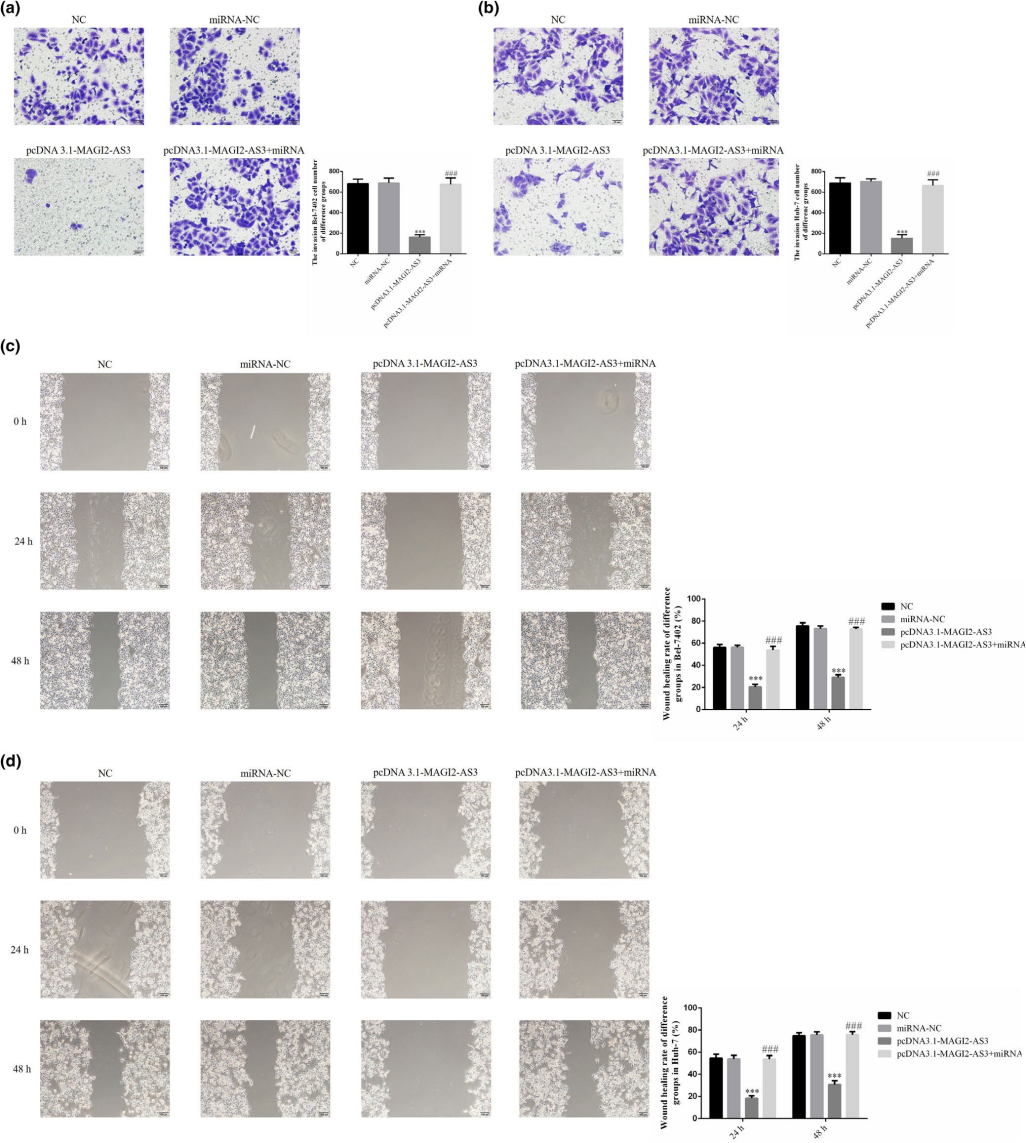
miRNA‐23a‐3p affect invasion and migration in Bel‐7402 and Huh‐7 cell lines. NC: The cell were treated with normal; miRNA‐NC: The cell were transfected with miRNA‐negative control; pcDNA3.1‐MAGI2‐AS3: The cell were transfected with pcDNA3.1‐MAGI2‐AS3; pcDNA3.1‐MAGI2‐AS3 + miRNA: The cell were transfected with pcDNA3.1‐MAGI2‐AS3 and miRNA‐23a‐3p (a) miRNA‐23a‐3p affect invasion Bel‐7402 cell number of difference groups by transwell assay (200×). ***: *p* <.001, compared with NC group; ###: *p* <.001, compared with pcDNA3.1‐MAGI2‐AS2 group. (b) miRNA‐23a‐3p affect invasion Huh‐7 cell number of difference groups by transwell assay (200×). ***: *p* <.001, compared with NC group; ###: *p* <.001, compared with pcDNA3.1‐MAGI2‐AS2 group. (c) miRNA‐23a‐3p affect Bel‐7402 cell wound healing rate of difference groups (100×). ***: *p* <.001, compared with NC group; ###: *p* <.001, compared with pcDNA3.1‐MAGI2‐AS2 group. (d) miRNA‐23a‐3p affect Huh‐7 cell wound healing rate of difference groups (100×). ***: *p* <.001, compared with NC group; ###: *p* <.001, compared with pcDNA3.1‐MAGI2‐AS2 group

The Transwell assay revealed that the number of invading Bel‐7402 and Huh‐7 cells was significantly lower in the pcDNA3.1‐MAGI2‐AS3 group than in the NC group (*p* <.001, Figure 6a,b). After the cells were co‐transfected with miRNA‐23a‐3p, the number of invading cells in the pcDNA3.1‐ MAGI2‐AS3 + miRNA group was significantly increased compared with the pcDNA3.1‐MAGI2‐AS3 group (*p* <.001, Figure 6a,b). Moreover, the wound healing assay revealed that the wound healing rates in Bel‐7402 and Huh‐7 cells at 24 and 48 hr were significantly lower in the pcDNA3.1‐MAGI2‐AS3 group compared with the NC group (*p* <.001, Figure 6c,d). After the cells were co‐transfected with miRNA‐23a‐3p, the wound healing rates in the pcDNA3.1‐MAGI2‐AS3 + miRNA group were significantly higher compared with the pcDNA3.1‐MAGI2‐AS3 group (*p* <.001, Figure 6c,d).

### 3.7 Effects of miRNA‐23a‐3p on relative protein expression and p‐AKT nuclear localization

**FIGURE 7:**
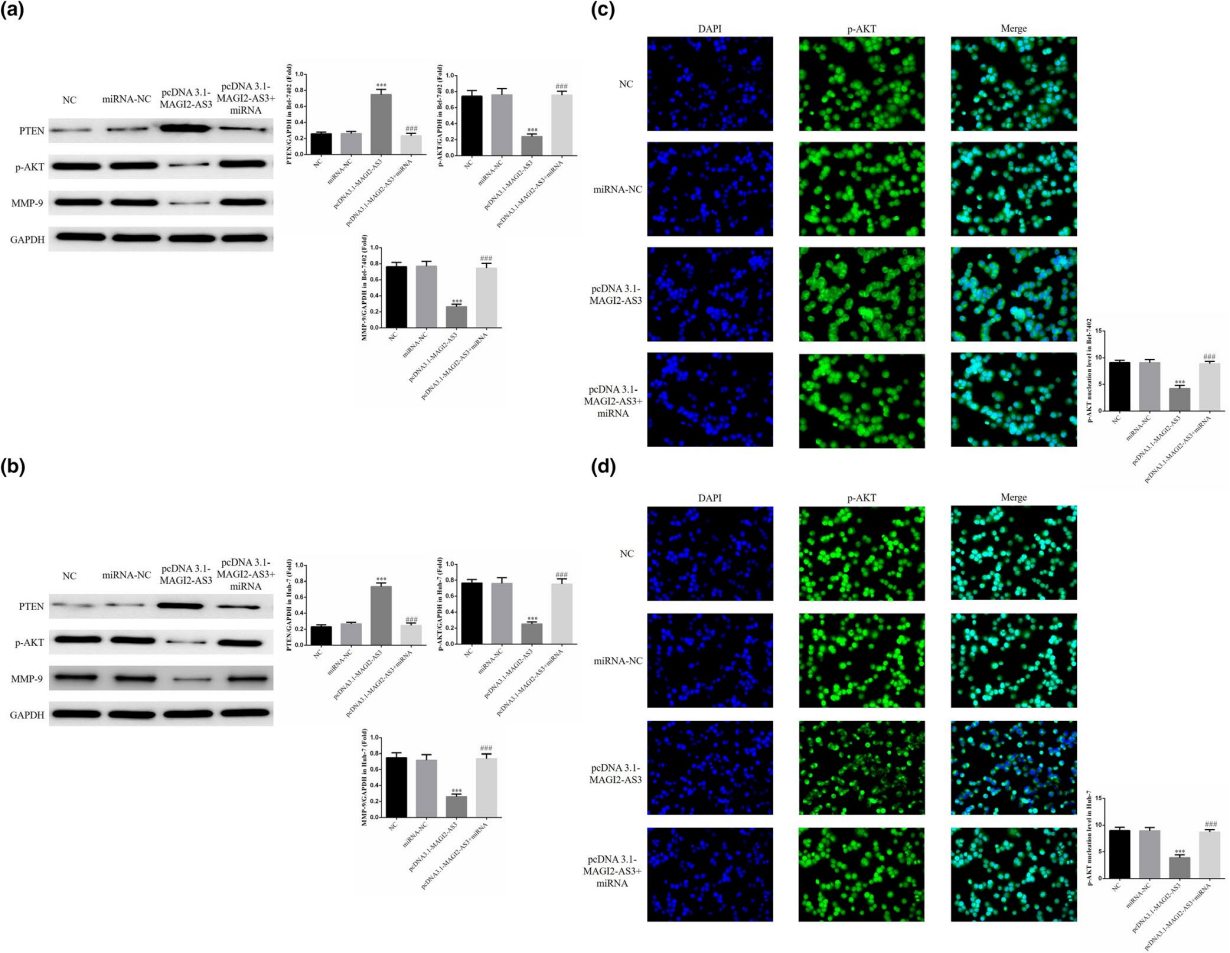
Effects of miRNA‐23a‐3p on Related Proteins and p‐AKT Nucleation. NC: The cell were treated with normal; miRNA‐NC: The cell were transfected with miRNA‐negative control; pcDNA3.1‐MAGI2‐AS3: The cell were transfected with pcDNA3.1‐MAGI2‐AS3; pcDNA3.1‐MAGI2‐AS3 + miRNA: The cell were transfected with pcDNA3.1‐MAGI2‐AS3 and miRNA‐23a‐3p. (a) miRNA‐23a‐3p affect relative proteins in Bel‐7402 by WB assay. ***: *p* <.001, compared with NC group; ###: *p* <.001, compared with pcDNA3.1‐MAGI2‐AS2 group. (b) miRNA‐23a‐3p affect relative proteins in Huh‐7 by WB assay. ***: *p* <.001, compared with NC group; ###: *p* <.001, compared with pcDNA3.1‐MAGI2‐AS2 group. (c) miRNA‐23a‐3p affect p‐AKT nuclear volume in Bel‐7402 cell. ***: *p* <.001, compared with NC group; ###: *p* <.001, compared with pcDNA3.1‐MAGI2‐AS2 group. (d) miRNA‐23a‐3p affect p‐AKT nuclear volume in Huh‐7 cell. ***: *p* <.001, compared with NC group; ###: *p* <.001, compared with pcDNA3.1‐MAGI2‐AS2 group

WB was employed to detect the relative protein expression. In the Bel‐7402 and Huh‐7 cell lines, the PTEN protein expression was significantly higher, and the p‐AKT and MMP‐9 expression was significantly lower in the pcDNA3.1‐MAGI2‐AS3 group compared with the NC group (*p* <.001, Figure 7a,b). After the cells were co‐transfected with miRNA‐23a‐3p, PTEN protein expression was significantly lower, whereas p‐AKT and MMP‐9 expression was significantly higher in the pcDNA3.1‐MAGI2‐AS3 + miRNA group compared with the pcDNA3.1‐MAGI2‐AS3 group (*p* <.001, Figure 7a,b). Cell immunofluorescence experiments revealed that in Bel‐7402 and Huh‐7 cells, nuclear localization of p‐AKT protein was significantly lower in the pcDNA3.1‐MAGI2‐AS3 group compared with the NC group (*p* <.001, Figure 7c,d). After the cells were co‐transfected with miRNA‐23a‐3p, the nuclear localization of p‐AKT protein was significantly higher in the pcDNA3.1‐MAGI2‐AS3 + miRNA group compared with the pcDNA3.1‐ MAGI2‐AS3 group (*p* <.001, Figure 7c,d).

### 3.8 Correlations among MAGI2‐AS3, miRNA‐23a‐3p, and PTEN

**FIGURE 8:**
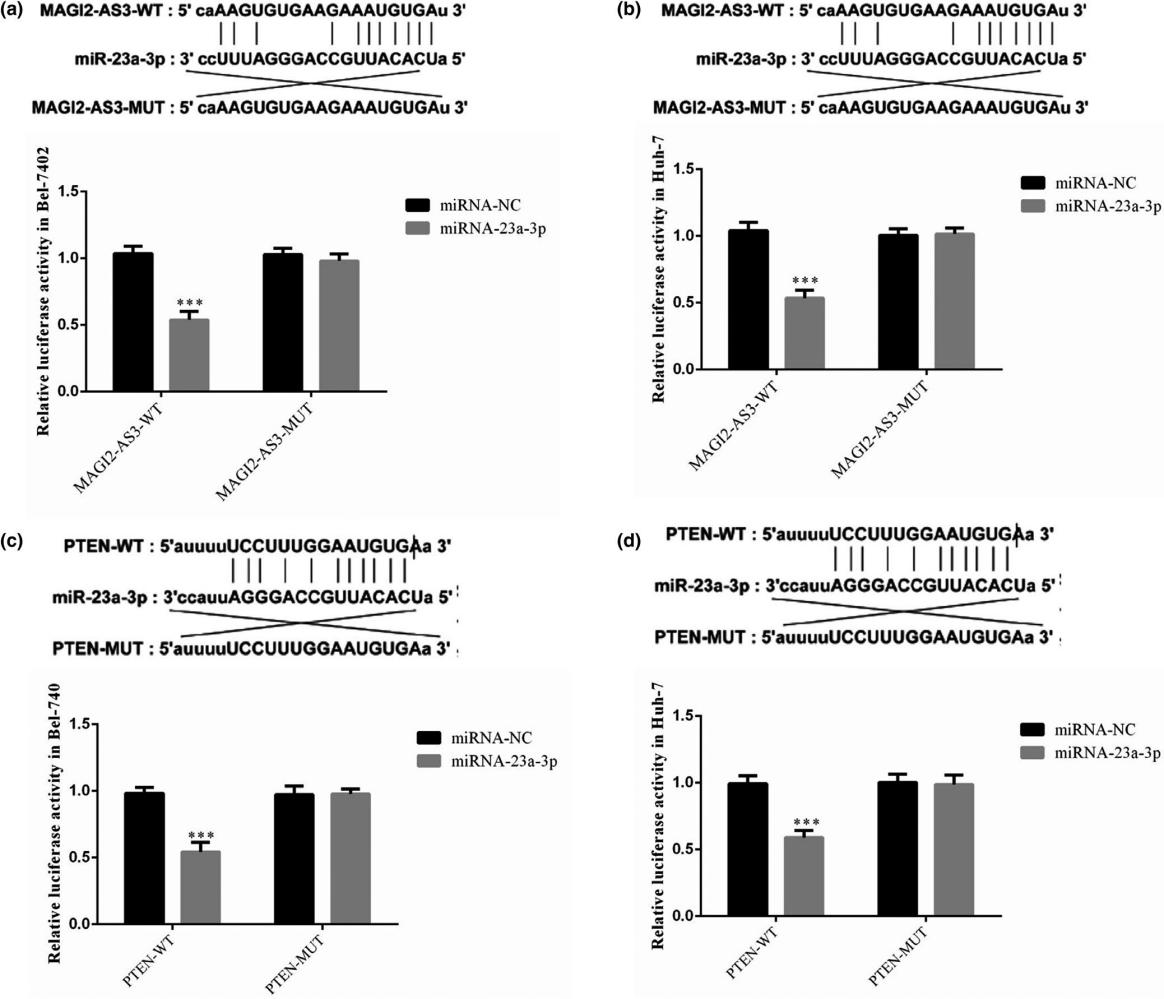
Correlations between MAGI2‐AS3, miRNA‐23a‐3p, and PTEN. (a) Correlation between miRNA23a‐3p and MAGI2‐AS3 in Bel‐7402 cell line. ***: *p* <.001, compared with miRNA‐NC. (b) Correlation between miRNA23a‐3p and MAGI2‐AS3 in Huh‐7 cell line. ***: *p* <.001, compared with miRNA‐NC. (c) Correlation between miRNA23a‐3p and PTEN in Bel‐7402 cell line. ***: *p* <.001, compared with miRNA‐NC. (d) Correlation between miRNA23a‐3p and PTEN in Huh‐7 cell line. ***: *p* <.001, compared with miRNA‐NC

A dual‐luciferase assay revealed that in Bel‐7402 and Huh‐7 cells, MAGI2‐AS3 could target the regulation of miRNA‐23a‐3p (Figure 8a,b). It was also found that in Bel‐7402 and Huh‐7 cells, miRNA‐23‐3p could target the regulation of PTEN (Figure 8c,d).

## 4 DISCUSSION

LncRNAs regulate gene expression by regulating RNAs at various levels (Kopp & Mendell, 2018; Slaby et al., 2017). Recent studies have reported that lncRNAs are widely involved in various biological processes and that the abnormal expression of lncRNAs is related to numerous diseases, including cancer (Lin & Yang, 2018). LncRNA SNHG20 enhances the proliferation and invasion by regulating ADAM10 (Guo et al., 2018). The dysregulation of lncRNA expression has been confirmed in numerous tumor types, suggesting that they play key roles in tumorigenesis. MAGI2‐AS3 is a type of lncRNA, and some studies have found a correlation between MAGI2‐AS3 and cancer development (Xu et al., 2017; Zheng & Liu, 2017). MAGI2‐AS3 also exerts effects on the biological activities of hepatic cancer cells (Jiang et al., 2016), but the effects and mechanisms of MAGI2‐AS3 in HCC treatment are still unclear.

In the present study, we evaluated lncRNA expression in HCC and adjacent nontumor tissues *via* chip assay. The results revealed that the lncRNA MAGI2‐AS3 was expressed at a low level in liver cancer tissues. RT‐qPCR showed that MAGI2‐AS3 gene expression was significantly lower in liver tumor tissues. The median expression of MAGI2‐AS3 in cancer tissues could be used to divide the patients into low‐ and high‐expression groups. A clinicopathologic analysis revealed that patients in the MAGI2‐AS3 high‐expression group had advantages in terms of tumor size, TNM stage, and metastasis compared with those in the MAGI2‐AS3 low‐expression group. Moreover, the follow‐up results revealed that the survival rate of patients was higher in the MAGI2‐AS3 high‐expression group than in the MAGI2‐AS3 low‐expression group. Further cell biological studies were conducted to explore how MAGI2‐AS3 functions in liver cancer.

In the in vitro cell experiment, after MAGI2‐AS3 was transfected into liver cancer cells, the biological activities (proliferation, invasion, and migration) of the liver cancer cells Bel‐7402 and Huh‐7 were significantly inhibited. It was also found that the level of miRNA‐23a‐3p expression was significantly inhibited by MAGI2‐AS3. A dual‐luciferase target experiment revealed that MAGI2‐AS3 could target the regulation of miRNA‐23a‐3p.

The expression of P13K/AKT signaling is dysregulated in a variety of human tumors. The activation of AKT by P13K phosphorylation promotes cell proliferation, invasion, and migration and inhibits apoptosis, which is the core effect of this pathway (Germano et al., 2019; Lee & Pandolfi, 2019; Xi et al., 2019). PTEN is a significant tumor suppressor gene that can negatively regulate P13K/AKT signaling, whereas the absence of its expression can lead to tumor progression, poor prognosis, and lymph node metastasis (Li et al., 2019; Song et al., 2019; Xi et al., 2019). PTEN dephosphorylates P13K and disables the activation of AKT, thereby inhibiting P13K/AKT signaling. For this reason, most researchers call this axis the P13K/PTEN/AKT signaling pathway (Zheng & Liu, 2017; Zheng et al., 2019; Zhu et al., 2019). In this study, after the transfection of MAGI2‐AS3 into liver cancer cells, as the biological activity of the liver cancer cells weakened, the expression of PTEN protein was significantly increased. However, its downstream p‐AKT activity and nuclear localization were significantly inhibited. Further molecular bioassays revealed that MAGI2‐AS3 overexpression activated PTEN, which might be related to the inhibitory effect of MAGI2‐AS3 on miRNA‐23a‐3p. A dual‐luciferase assay revealed that PTEN was a target protein of miRNA‐23a‐3p.

The activation of AKT enhances the proliferation of tumor cells and suppresses apoptosis (Golubnitschaja et al., 2016; Liang et al., 2017). Moreover, activated p‐AKT can also activate downstream matrix metalloproteinases (MMPs) by increasing nuclear localization. This in turn leads to enhanced invasion and migration in cancer [34,35]. MMP‐9, a member of the MMP family, can specifically degrade type IV collagen. The inhibition of MMP‐9 expression in liver cancer can reduce the invasiveness and migration of cancer cells [36]. The findings of this study confirmed that when MAGI2‐AS3 was overexpressed by transfection into cells, it could effectively depress MMP‐9 expression and thus suppress both invasion and migration.

In summary, the lncRNA MAGI2‐AS3 was decreased in HCC tissues. In vitro cell experiments confirmed that MAGI2‐AS3 overexpression by liver cancer cell transfection could effectively inhibit their biological activity. The underlying mechanism might involve the miRNA‐23a‐3p/PTEN axis. Therefore, MAGI2‐AS3 can be used as a promising tool for diagnosing and treating hepatocellular carcinoma.

## CONFLICT OF INTEREST

We have no conflict of interest in the authorship and publication of this article.

## STUDIES INVOLVING HUMAN SUBJECTS

The study conforms to the Declaration of Helsinki, US, and/or European Medicines Agency Guidelines for human subjects.

## STUDIES INVOLVING ANIMAL OR HUMAN SUBJECTS

A statement that the study's protocols and procedures were ethically reviewed and approved by a recognized ethical body (provide its name); US authors should attest to compliance with US National Research Council's Guide for the Care and Use of Laboratory Animals, the US Public Health Service's Policy on Humane Care and Use of Laboratory Animals, and Guide for the Care and Use of Laboratory Animals. UK authors should conform to UK legislation under the Animals (Scientific Procedures) Act 1986 Amendment Regulations (SI 2012/3039). European authors outside the UK should conform to Directive 2010/63/EU. All patients signed informed consent.
